# Exploring the Impact of a Global Pandemic (COVID-19) on Factors Impacting the Resilience of Top-Tier London Hockey Players

**DOI:** 10.1155/2023/5346846

**Published:** 2023-07-07

**Authors:** Philippa Boag Sharland, Justin Haroun, Ayazullah Safi

**Affiliations:** ^1^Centre for Resilience, School of Life Sciences, University of Westminster, London, UK; ^2^Department of Public Health, Centre for Life and Sport Science (C-LaSS) Birmingham City University, Birmingham, UK

## Abstract

**Introduction:**

The cessation of all professional and amateur sport due to the COVID-19 pandemic had a dramatic effect on the mental and physical capacity of the United Kingdom populace, but its impact was arguably felt more deeply by the athletic population. Thus, this research explored which limiting factors were experienced by team hockey players during the national lockdowns (1.0–3.0) with the objective of enabling coaches and team management to better support and protect players' physiological and psychological resilience in return to play.

**Methods:**

Data were collected over 12 weeks during the 3rd UK lockdown (March 2021) from two top-tier London clubs. Hockey players (*n* = 63) completed an online questionnaire that included validated tests for self-compassion, sport motivation, and a custom open-ended style qualitative questionnaire on nutrition and lifestyle behaviour. Mean self-compassion, motivation scores, and common indicative limiting factors were evaluated and ranked according to significance.

**Results:**

High “rebound resilience” was found with low amotivation scores (*m* = 8.33) and strong affinity for their sport identifying with the statement “because participation in my sport is an integral part of my life” with correspondingly high integrated regulation scores (*m* = 21.43). Participants' self-compassion showed the highest scores in mindfulness (*m* = 3.66) and lowest in self-kindness (*m* = 2.84) indicating a common trait in athletes for self-criticism. The highest limiting factor was cited as “no social outlets, social interactions, or seeing friends and family”.

**Conclusion:**

This study revealed the critical role that social connectedness plays in promoting resilience and enhancing motivation in return to play during extremely challenging circumstances. When the social outlet is absent, enhancing resilience factors with mindfulness, self-compassion, and the creation of a more facilitative environment where player welfare takes priority are potential strategies to support players when they are unable to participate in their sport.

## 1. Introduction

The national lockdowns in the United Kingdom (1.0: March 2020; 2.0: October 2020; 3.0: January 2021) were employed to curtail the spread of a novel virus transmitting globally (COVID-19). The measures introduced by the UK Government reduced face-to-face contact and limited social and physical contact. “Social distancing” and the creation of “bubbles” meant that typical support networks were not available to individuals. Scientific research into the social impact of the pandemic on the people, households, and communities in the Great Britain, via the Opinions and Lifestyle Survey (OPN), monitored anxiety levels in a representative sample (*n* = 4,000) as one pertinent indicator of well-being [1]. Adults stated that their well-being was being affected by COVID-19 with anxiety rising to 57% on 10th–14th February 2021 comparable to levels of 53% during the first national lockdown, 27th March–5th April 2020 [1]. In a study of Austrian citizens (*n* = 902) during the same period (23rd April–30th April 2020) when comparable stay-at-home lockdown measures were in place, social connectedness was seen to potentially protect individuals from adverse physical and mental health outcomes and promote resilience [2].

Theories studying human behaviour and resilience are numerous [3–5], including mental, physical, social, and ecological effects in challenging environments, but this research set out to examine resilience in an athletic population subset predisposed to exercise; Vlachopoulos et al. [6] referred to it as having an “exercise identity” shown by measurement of self-reported minutes of weekly exercise, number of weeks of exercise participation, perceived exertion during exercise, muscular endurance, percentage of body fat, and fitness levels [7, 8]. Previous studies demonstrate a positive correlation between physical activity (PA) and health with exercise, triggering chemicals in the human body that improve cognitive functioning, supporting learning,long-term memory, and protecting the brain from neurodegenerative disease as well as stimulate new cell growth in the hippocampus [9, 10]. The link between PA and mental well-being before and after the global pandemic [11, 12] is equivocal. Data on levels of PA during the pandemic underlined a mixed response in the UK populace [13]. The direction of change showed both a 26.5% increase and a 25.2% decrease in PA [13]. The mental health of elite athletes has specifically come under scrutiny leading to a debate about when PA, at higher levels of volume and intensity, shifts from offering positive mental health protection to driving high-performance anxiety [14].

**Figure 1:**
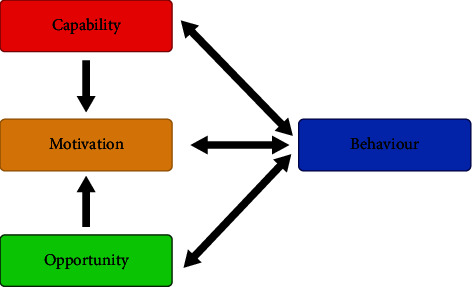
The COM-B model for behaviour change [15].

The COM-B model for behaviour change in Figure 1 [15] identifies three factors that need to be present for behavioural change to be enacted: capability, opportunity, and motivation. A targeted review of motivational behaviour within sport revealed a notable absence of research exploring factors impacting return to play (RTP) after physical, psychological, or societal change such as injury, mental incapacity, or national quarantine [16–18].

A study by Sports England [19], mapping the COM-B behavioural change model on UK data, highlighted perceived “opportunity” to be physically active dropped by 1.5% but “capability” and “motivation” remained unchanged. With the complete cessation of all professional and amateur sport during the three UK lockdowns, the impact on athletes, especially on elite sportswomen, is believed to have been substantial [20–22].

During the UK lockdown, social and environmental factors were radically altered for the individual and most social groups were no longer accessible. Social identity theory [23] reinforces behavioural change as dependent on the existing environment and how individuals are observed by themselves and perceived by others within a group setting. When the individual typically forms part of a team, where culture and practice could be different from the norm, lockdown restrictions offered a unique opportunity to explore competing behaviours and rank limiting factors in order of significance under the assumption of self-determination theory (SDT [24], where the fulfilment of basic needs for autonomy, competence, and relatedness is seen to typically regulate behaviour.

The aim of this research was to examine the limiting factors experienced by top-tier hockey players in relation to nutrition, motivation levels, and mental well-being during the national lockdowns (1.0–3.0) with the objective of enabling coaches and team management to better support and protect players physiologically and psychologically in RTP. The sporting environment is continually challenging its players to be the best and maintain a competitive edge but there is mounting evidence to suggest this cannot be achieved merely by enhancing physiological capacity [25]. Therefore, psychological growth is also regarded as important [26]. The variables under review included resilience or coping [27], motivation [28], and self-compassion [5]. By overlaying the COM-B model of behaviour framework, the objective was not only to explore limiting factors during lockdown for sports players but to investigate how extreme environments that reduce capacity can challenge motivational precepts and facilitate opportunity in the form of positive enablers for future development.

## 2. Methods

### 2.1. Participants

Players were recruited using purposive sampling from three top-tier hockey clubs in London (*n* = 687) by contacting club gatekeepers during lockdown. Validated questionnaires exploring individual motivation and compassion levels using a Sports Motivation Scale and Self-Compassion Scale (SMS-6 [29] and SCS-SF [30]) and a customised questionnaire on nutrition and lifestyle behaviour were emailed to the players. The study sample (Table 1) included completed data from 63 participants from two London clubs, one competing in the national premier league. The gender split was 27 male and 36 female respondents. The mean age of participants was 32 years (SD = 9.5 and range = 18–70 years) with 60 (95.2%) playing for 10+ years. 5 hockey coaches were additionally recruited via email from the premier league club whose players recorded the highest response rate.

### 2.2. Methodology

The research used a questionnaire combining validated psychometrics and open questions. Specifically, a quantitative validated short-form Self-Compassion Scale (SCS-SF) [30] adapted from [31], a validated Sports Motivation Scale [32], in a revised shorter form (SMS-6) [29] and a custom open-ended style qualitative questionnaire on nutrition and lifestyle behaviour developed by the author utilising recommended protocol [33–36]. Both the Self-Compassion Scale and Sports Motivation Scale were used in short form to not overburden the respondents with too many questions given multiple factors were being explored. The questionnaire commenced with a detailed summary of the research parameters followed by an opt-in for full written consent from each participant. Furthermore, a short online questionnaire was conducted with coaches to share any experiences of RTP and lockdown reflections. The Self-Compassion Scale short-form questionnaire measured participants' attitudes to the following, “How I typically act towards myself in difficult times,” and was used to assess at what level premier hockey team players engage in the cognitive, attentional, and emotional behaviour associated with self-kindness and mindfulness during a time of heightened pressure. The total mean scores for self-compassion were computed by calculation of the mean of subscale item responses including the reverse scores related to some items designed to measure lack of compassionate behaviour towards oneself—the negative subscale items of self-judgement, isolation, and overidentification [37]. The Sports Motivation Scale short-form questionnaire gauged participants' views toward the following question “Why do you practice your sport?” and was applied as a measure of participants' ongoing motivation toward their sport despite social restrictions and sport being banned. The Self-Compassion Scale used the 5-point Likert scale to apply a numerical measure against responses depending on how strongly they agreed or disagreed with the given statements with 1 being “almost never” and 5 “almost always.” The Sports Motivation Scale used the 7-point Likert scale with 1 being “does not correspond at all” and 7 being “corresponds exactly.” The concurrent qualitative lifestyle behaviour questionnaire was designed to add detail, depth, and context to the quantitative data and centred on key performance-related indicators for sports players' fitness, nutrition, and mental well-being [25], as described in the study by [14, 38].

### 2.3. Data Collection and Ethical Consideration

Phase 1: An online questionnaire answered anonymously by targeted hockey players in top-tier London clubs. The questionnaire was open for a twelve-week period (22nd March to 11th June 2021).

Phase 2: An online questionnaire with team coaches to add stakeholder perspective for a comprehensive rigorous study approach. The recruitment periods overlapped, and during an eight-week period, the coaches from the teams who provided the highest response rate to the player questionnaire were asked for feedback.

The noninvasive method of data collection, an online questionnaire, correlated with a low-risk project due to the limited health and safety exposure for its participants. Institutional ethical approval was gained from the University of Westminster prior to the data collection. The questionnaire sent to the participants was disseminated via the JISC online platform as it is fully GDPR compliant and certified to ISO9001 and ISO27001. Consent formed part of the questionnaire, and its completion acted as written informed consent.

### 2.4. Statistical Analysis

The data analysis was performed using the IBM SPSS Statistics version 26 at a 95% confidence interval [39]. Descriptive statistics were used to introduce the baseline data. The data were normally distributed (*p* < 0.5) for all 12 self-compassion (SCS-SF) and 22 motivation (SMS-6) questions. An independent *t*-test was performed to assess any significant gender differences.

### 2.5. Qualitative Analysis

Data from the custom open-ended style questionnaire was analysed utilising a deductive approach by inferring that the lockdown had a limiting effect on sports players who were no longer able to participate in their sport. Nutrition, fitness levels, and mental well-being were the key performance indicators being evaluated for association, behavioural change, and expectations. These indicators were selectively grouped using a thematic approach and further coded into subthemes based on the most cited factors by the respondents and then verified by a 2nd researcher. The ranking of limiting factors was considered within the framework of the COM-B Model for Behaviour Change which assumes capability, opportunity, and motivation need to be present for any behavioural change to be enacted.

## 3. Results

### 3.1. Quantitative Analysis of Self-Compassion Scale Short Form (SCS-SF [30])

The mean self-compassion scores with standard deviation are presented in Table 2 with a total self-compassion score for the sample (*m* = 3.08 and SD = 1.11). The scale had an acceptable level of internal consistency, as determined by Cronbach's alpha (*α* *=* 0.74) in Table 3. A proficient level of internal consistency differs depending on what source you refer to, although all recommended values are 0.7 or higher [40] (Kline, 2005). The Cronbach's alpha demonstrated a questionable fit for overidentification; however, if question 1 was deleted, it delivers a higher measurement (*α* *=* 0.82).

### 3.2. Quantitative Analysis of Sport Motivation Scale (SMS-6 [29])

The sport motivation mean scores with standard deviation for all 22 questions are presented in Table 4 with a total mean sports motivation score (*m* = 4.21 and *n* = 63). The scale had a high level of internal consistency, as determined by Cronbach's alpha (*α* *=* 0.84) in Table 5. There were 36 male and 27 female participants. Motivation mean scores were found to be comparable for male hockey players (*m* = 4.21 and SD = 0.82) and female hockey players (*m* = 4.21 and SD *=* 0.65). An independent *t*-test was conducted to examine motivation score differences between the sexes. No significant differences were found in motivation between male and female hockey players, *p*=0.002 and 95% CI (−0.38 to 0.38).

### 3.3. Qualitative Thematic Analysis of the Perceived Impact on Nutrition, Fitness Levels, and Mental Well-Being Nutrition

Nutritional themes of improvement, deterioration, and food access led to subthemes of time, quality, emotional eating, and diet adaptation (Table 6).

In this study, 23 (37.7%) respondents made perceived improvements to nutritional intake, with the same number, 23 (37.7%), claiming maintenance of their nutritional intake. 10 (16.7%) reported a perceived deterioration in their nutritional intake, while 5 (8.2%) self-reported both improvement and deterioration at different times of stress during the three lockdown periods.

### 3.4. Physical Fitness

Performance themes of adaptation and physiological impact led to subthemes of fitness levels, body composition, energy levels, cognitive focus, and sleep patterns (Table 7).

Results show that 54 (87.1%) participants noticed physiological changes with 8 (12.9%) perceiving no change at all.

### 3.5. Mental Health

Well-being themes of social restriction, working from home, and coach and club support led to subthemes of self-criticism, behavioural change, and communication (Table 8).

New behavioural strategies in relation to maintaining fitness, eating more mindfully, and improving self-care were reported as beneficial changes during lockdown, whereas common detrimental changes listed were increased screen-time, reduced exercise, work-life imbalance, limited social interaction, and increased alcohol consumption and takeaway deliveries.

### 3.6. Coach Experience of Return to Play

Coach reflection themes of role change, team contact, and return to play led to subthemes of focus, making the most of it, mental health, and physical fitness (Table 9).

Responses revealed mental health concerns for many of the players; however, motivation for RTP was high.

### 3.7. Limiting Factors

  These were explored using the COM-B Model of Behaviour (Figure 1)
  Capability (barriers ranked in descending order of importance) *n* = 11
  “Injuries and inability to treat them” (*n* = 6) | “COVID/bereavement” (*n* = 3)
  “Burnout/mental health” (*n* = 2).
  Motivation (barriers ranked in descending order of importance) *n* = 40
  “Boredom/lack of structure” (*n* = 14) | “lack of motivation/focus” (*n* = 12)
  “Personal stress” (*n* = 9) | other categories (*n* = 16)
  Opportunity (barriers ranked in descending order of importance) *n* = 125
  “No social outlets/social interactions/seeing friends and family” (*n* = 44)
  No gym access/formal hockey (*n* = 35) | WFH stress/workload/job insecurity
  (*n* = 31) | other categories (*n* = 15)

## 4. Discussion

This study seized upon the opportunity during the UK lockdowns to encapsulate the experience and perceptions of top-tier London hockey players in relation to the impact that the government restrictions had on their personal nutrition, physical fitness, and mental health before RTP. It was reported that two-thirds of participants experienced some level of disruption to their diet and lifestyle during the pandemic restrictions in a UK national survey [13]. Psychological resilience is defined as “the role of mental processes and behaviour in promoting personal assets and protecting an individual from the potential negative effect of stressors” [41]. The immediate environment and its careful management were found to be pivotal in protecting athletes from negative consequences.

### 4.1. Nutrition

Reducing the nutritional intake to compensate for changes in energy expenditure was feasibly indicative of an “exercise identity” [7]. Food was commonly seen as an emotional connection with the consumption of “comfort food” out of boredom, tiredness, or working from home (WFH) with immediate access to snacks. There was a strong aspiration to enhance nutritional balance, safeguard consistency, and adopt a more mindful attitude toward nutritional intake despite diminished structure with WFH. The improvers highlighted that consumption of home-cooked food, frequently from scratch, delivered better quality nutrition reporting it as a positive behavioural change. A small minority of respondents took the opportunity to explore different fuelling strategies such as intermittent fasting to monitor how it affected energy levels. The majority who recognised deterioration were conscious of a behavioural change and perceived it as short-term with the acknowledgment that when restrictions were lifted, they were inherently capable of reverting to a regular routine. The language used evoked a sense of robustness and mental resilience among those who had suffered temporary setbacks. The highest mean score of mindfulness on the SCS-SF [30] indicates a consciousness and nontendency to overreact to potentially overwhelming circumstances. The lowest mean score for self-kindness suggests that this group can be overly critical. This could imply that an opportunity exists to further improve self-kindness by reducing high levels of self-criticism which is a commonly identified trait of competitive athletes [26] by focusing on ways to sustain and even increase mindfulness scores to protect athletes when presented with challenging circumstances in the future.

#### 4.1.1. Practical Implications

Knowledge, which forms an important component of intrinsic motivation [28], offers better protection for players against future injury and a mental readiness for RTP. There was adequate awareness of the need for athletes to fuel their bodies appropriately with reference to food for recovery; however, the extent to which energy systems linked to physical performance was not widely acknowledged. The openness to learn new skills and adopt new habits in respect to nutritional intake signals a potential opportunity to further educate this group and maximise physical performance. The small margin between victory and loss is substantiated [42], and in isolation, athletes are more vulnerable to targeting by performance-related supplement companies. These can be dangerous for many reasons: safety, legality, and lack of proven efficacy [43]. Enlisting the support of nutrition sports performance professionals (IOPN and ISEH) to provide evidence-based research presents a plethora of opportunities for beneficial improvements and personalised management of fuelling the body for optimal results and protecting the body in times of stress.

### 4.2. Physical Fitness

Participants who experienced perceived physiological changes, frequently mentioned the impact on concentration levels, gains in body mass (10–20% quoted), and a lack of energy, repeatedly described as lethargy. This was often associated with WFH, closed gyms, training sessions being prohibited, and a reduced nutritional intake. However, approximately one-third of this cohort experienced enhanced body composition, namely, weight loss with muscle gain and improved energy levels. This was attributed to having more time to reflect, plan, and initiate improved self-care. Predominantly, respondents highlighted adaptive training strategies and demonstrated autonomous control of personal fitness, yet suffered frustrations in relation to cardiovascular and strength training fraught with time and space constraints and a lack of equipment in the home. The majority missed the outdoor training sessions, the reduced variety due to gyms, pitches, and pools being closed and the resultant loss in PA intensity. However, this was dependent upon the level of play, self-motivation, and interest in alternative training activities such as cycling or running. Many participants acknowledged that a proposal to build up fitness and strength for RTP would have been ideal but difficult to plan, given the uncertain timing of lockdown; however, teams commended coach-organised physio sessions, Strava running competitions, 1 : 1 Zoom calls, and optional online high-intensity interval training (HIIT). Reflecting on the support received in lockdown, multiple participants would have appreciated regular check-ins and more structured at-home training schedules with hockey-specific movement exercises and strength sessions. It was commonly anticipated that coaches who enforced a strict fitness protocol however would have been met with active resistance. There was a strong feeling that personal fitness could be managed autonomously though they would have benefited from steady encouragement and missed the competitive edge offered by the team training sessions. This study found no difference in the motivation levels between gender; however, Lautenbach et al. [44] found from a study on amateur and professional athletes during the lockdown from April to mid-May 2020 in Germany that there were no differences according to the type of sport (individual vs. team sport) but the motivation to train was found to be lower in females than in males (*p*=0.029). In addition, differences in emotional state, perceived stress, and personality variables (orientation to happiness, volition) were found between athletes who stated that they were less motivated to train compared to athletes who reported no changes in motivation. Even though athletes in Lautenbach's study received emotional support, organised themselves via routines and schedules, and trained using online tools, they predominantly stated that they wished that their coaches had supported them more. Vlachopoulos et al. [6] found that the participation level was a strong predictor of motivation which we were not able to explore in any detail in our study as most respondents played at a comparable high level and had 10+ years' experience.

#### 4.2.1. Practical Implications

Two-way communication, on social restriction updates, logistics and a roadmap to RTP, and positive reinforcement around training, goals, and expectations for the next season would have been appreciated while being mindful to show compassion toward individual circumstances. The overwhelming response to fitness level assessment was that it could be managed personally nevertheless access to hockey-specific skills training videos and data on the high-level strategic play would have been a beneficial enabler. This feeds into the intrinsic motivational need for stimulation. Creating a facilitative environment to encourage developmental feedback on how to improve, praising accomplishments, supporting learning, and building trust is effective in cultivating resilience [41]. The importance of resilience is illustrated in a study where it emerged as one of the four major themes regarded as central to an individual football player's success [45]. Another physiological positive that could be capitalised on by coaches postpandemic is the fact that in team sports traditionally there are minimal times in the sporting calendar when a player can have a prolonged period of complete recovery from specific training, injuries, and competition demands [22].

### 4.3. Mental Health

Crucial in the sporting context is how to support athletes in reaching their full potential in a nonmaladaptive manner, especially given the “athlete condition” [14], where perfectionist's character traits are exacerbated by performance-driven anxiety [46]. Athlete success is often viewed as being achieved at a personal cost. The development of emotional self-control is one key determinant in the path to peak performance [46] and the adoption of self-compassion which involves the capacity to bounce back and self-soothe after experiencing emotions relating to failure, suffering, or personal inadequacy. The ability to re-energise and motivate oneself is believed to be a learned behaviour that can be encouraged through the development of compassion for others, and improved self-compassion can sustain our compassion for others [47]. Increased levels of self-compassion in general have been shown to alleviate emotional, cognitive, and physical exhaustion [48]. Individuals with a higher sense of self-compassion (self-rated) were found to have decreased levels of cortisol which were associated with a greater ability to gain emotional control and self-soothe when stressed [49]. Its importance cannot be overstated when sports players are returning postinjury, post-trauma, or postquarantine. The total self-compassion score result for the hockey players (*m* = 3.06) revealed a moderate score [31]. Scores in self-kindness (*m* = 2.84) were the lowest of all the six constructs with mindfulness being the highest (*m* = 3.66). The highest mean self-compassion score (3.71 ± 0.85) was observed for question 3, “When something painful happens, I try to take a balanced view of the situation” (mindfulness). The lowest mean self-compassion score (2.62 ± 1.02) was observed for question 6, “When I am going through a very hard time, I give myself the caring and tenderness I need” (self-kindness). A greater life satisfaction is indicated by higher mindfulness scores [37], which is a positive reflection on the ability of the participants in this study to take a balanced approach to adverse situations. Lack of “opportunity” to interact socially accounted for the highest perceived disruption factor listed (*n* = 44); however, participants commented on an improved sense of community in their locality. Indeed, the absence of social activities could lead to a higher appreciation and strengthening of key relationships such as the player-coach contact when sport returned [50]. This current study highlighted that the participants, however strongly intrinsically motivated, have a critical requirement for social interaction and see their sport, hockey, as a life enabler with 36% choosing to correspond exactly with the statement “For the excitement I feel when I am really involved in the activity” and 34.9% engaging exactly with the statement “Because participation in my sport is an integral part of my life.” Many studies have found that females reported higher levels of perceived stress during lockdown [51] but the reasons have not been conclusive. Bowes et al. [20] exposed a potential source of anxiety for female athletes, especially elites, believing them to be in fear of suffering postpandemic-reduced sponsorship and/or match fees and experiencing increased burden due to imbalanced child-care responsibilities during the lockdown. International studies [2, 44] have shown differences in stress variables dependent not only on the athletes' country of residence but also on sport type, the frequency of cancelled competitions, and overall concern for the future of their sport.

#### 4.3.1. Practical Implications

Psychological training could be employed and monitored to nurture the resilience among athletes that lead to improved consistency in sporting performance postfailure or setbacks. Clubs and academies could benefit from employing staff trained in player welfare and with the mental health literacy to promote an environment that celebrates inclusivity and positive pathways and with the responsibility of overseeing a support system [14]. The involvement of all stakeholders in the drive to incorporate the development of self-compassion in training and competition routines can help identify weaknesses and vulnerabilities and therefore offer opportunities for improvement and support [26]. For true success, it must be felt to be relevant to the athlete. Establishment of a suitable forum by management teams to cultivate help-seeking behaviours which include identifying and removing barriers and stigma and promoting continual a two-way flow of communication since participants did not mention that coach or hockey club management interactions added pressure or anxiety. A mentor scheme could also initiate improved empathy between various participation levels and age groups to enhance the development process. This is supported by the challenge and support concept [52] that commends the instilling of accountability and responsibility in individuals where expectations are high in relation to goal achievement, consistency, and winning tournaments. It is important to note that if too much challenge and not enough support are imposed then the unrelenting environment will compromise well-being. Conversely, if too much support and not sufficient challenge are provided, then a comfortable environment will not enhance performance [41].

## 5. Strengths and Limitations

This study was delivered in the framework of living and researching under the same social restrictions as the athletes during COVID-19 lockdowns in the UK. It may have been conducted via in-person focus groups if the restrictions had not been in place conceivably delivering deeper insight through skillful questioning (motivational interviewing) [53] and direct exposure to team dynamics. However, parity existed between the researcher and the respondents with the UK populace living under the same strict protocol. A factor to consider is that questionnaires are self-reported and thus open to respondents underplaying or overplaying responses, biased by socially desirable responding (SDR) [54]. Online questionnaires do not always capture the limbic behaviour and practices of the respondents and are constructed to deliver data based on rational thought and cognitive decision-making which may have been compromised during the UK national lockdowns. No cause-effect relationships can be drawn from this study. The sample size was small (*n* = 63) and the data collected in London so for increased applicability, further research would be required nationwide, comprising players in different leagues with a higher ethnic diversity since the study participants were predominantly Caucasian (92.1%). Research into gender discrepancies would be beneficial to provide additional insight into the extent to which inequalities may have been exacerbated by the global pandemic. The study's applicability to other team sports beyond hockey should be considered. The nonavailability of top-tier senior coaches due to Olympic involvement [55] meant that Junior Club coaches were approached for lockdown reflections on how they supported teams throughout the UK lockdowns. However, this added a different dimension to the study with the potential for learning from the ground up within a sports organisation. A fundamental strength of the study was the calibre of the participants.

## 6. Conclusion

The most indicated limiting factors for top-tier London hockey players during the UK lockdowns were focused on “opportunity” as to be expected with all play suspended and leisure facilities closed but “capability” and “motivation” levels held up (COM-B Model of Behaviour) [15]. This demonstrates a considerable “rebound resilience” with high integrated regulation and low amotivation. However, the most limiting factor quoted, abovementioned cessation of sport and working from home stress, was the absence of social interactions with friends, family, and teammates. This supports the basic psychological needs theory [56] that relatedness or social connectedness, identified as a critical component of intrinsic motivation, is essential when building resilience and improving coping mechanisms. Mindful self-compassion (MSC) is emerging as a beneficial tool to enhance personal capacity and growth mindset. Developing a positive culture that values the importance of social connectedness and respects player welfare requires a breadth and depth of commitment from management, technical and support staff, coaches, players, and parents. For resilience to be further nurtured, it takes time, robust relationships, a safe environment that encourages sensible risk-taking, healthy competition, and recognition and celebration of successes [41]. As noted by Morgan et al. [57], individual resilience does not necessarily translate to group-level resilience in team sports and this may require the promotion of “collective efficacy” as proposed by Bandura [58] (p. 76), whereby a shared belief in a team's ability to perform a task is better as one unit. This shows that relationships appear to be an important factor for resilience which means not only is the player-coach relationship important but also relationships at a team level and furthermore the social interactions with all stakeholders within the facilitative environment in which they exist.

## Figures and Tables

**Table 1:** Gender and playing level data.

Total number and percentage of participants	Gender		Number and percentage of participants by playing level				
	Male	Female	Noncompetitive recreational	Local	Regional	National	International
63 (100%)	27 (43%)	36 (57%)	5 (7.9%)	22 (34.9%)	13 (20.6%)	13 (20.6%)	10 (15.9%)

**Table 2:** Self-Compassion Scale (short form) mean score, reverse mean, and standard deviation (SD).

Self-compassion questions	Mean	Reverse mean	SD
When I fail at something important to me, I become consumed by feelings of inadequacy (*R*)	3.06	2.94	1.08
I try to be understanding and patient towards those aspects of my personality I do not like	3.06	3.06	1.08
When something painful happens, I try to take a balanced view of the situation	3.71	3.71	0.85
When I am feeling down, I tend to feel like most people are probably happier than I am (*R*)	2.83	3.17	1.34
I try to see my failing as part of the human condition	3.19	3.19	0.97
When I am going through a very hard time, I give myself the caring and tenderness I need	2.62	2.62	1.02
When something upsets me, I try to keep my emotions in balance	3.60	3.60	1.10
When I fail at something important to me, I tend to feel alone in my failure (R)	3.30	2.76	1.13
When I am feeling down, I tend to obsess and fixate on everything that is wrong (*R*)	3.16	2.84	1.25
When I feel inadequate in some way, I try to remind myself that feelings of inadequacy are shared by most people	3.10	3.10	1.09
I am disapproving and judgmental about my own flaws and inadequacies (*R*)	3.27	2.73	1.19
I am intolerant and impatient towards those aspects of my personality I do not like (*R)*	2.73	3.27	1.18
Total	3.14	3.08	1.11

(*R*) = Likert scale scores reversed for total self-compassion mean score 1 = 5, 2 = 4, 3 = 3, 4 = 2, and 5 = 1.

**Table 3:** Self-Compassion Scale (short form) mean score and Cronbach's alpha reliability score.

Self-compassion subscale	1st question	2nd question	Mean	Cronbach's alpha
Self-kindness (Q. 2 and 6)	3.06	2.62	2.84	0.43
Common humanity (Q. 5 and 10)	3.19	3.10	3.15	0.35
Mindfulness (Q. 3 and 7)	3.71	3.60	3.66	0.59
Self-judgement (Q. 11 and 12)	2.73^*∗*^	3.27^*∗*^	3.00	0.75
Isolation (Q. 4 and 8)	3.17^*∗*^	2.76^*∗*^	2.97	0.60
Overidentification (Q. 1 and 9)	2.94^*∗*^	2.84^*∗*^	2.89	−2.9
Total	3.13	3.03	3.08	0.74

^*∗*^Negative classification items where Likert scale scores were reversed for total self-compassion mean score.

**Table 4:** Sports Motivation Scale (short form) mean score and standard deviation (SD).

Number	Questions	Mean (SD)
1	For the excitement I feel when I am really involved in the activity	5.76
		(1.29)

2	Because it is part of the way in which I have chosen to live my life	5.56
		(1.29)

3	Because it is a good way to learn lots of things which could be useful to me in other areas of my life	4.06
		(1.52)

4	Because it allows me to be well regarded by people that I know	3.83
		(1.91)

5	I do not know anymore; I have the impression of being incapable of succeeding in this sport	1.94
		(1.19)

6	Because I feel a lot of personal satisfaction while mastering certain difficult training techniques	4.83
		(1.48)

7	Because it is absolutely necessary to do sports if one wants to be in shape	4.30
		(1.75)

8	Because it is one of the best ways, I have chosen to develop other aspects of my life	4.17
		(1.74)

9	Because it is an extension of me	5.25
		(1.65)

10	Because I must do sports to feel good about myself	4.59
		(1.78)
11	For the prestige of being an athlete	3.46
		(1.74)

12	I do not know if I want to continue to invest my time and effort as much in my sport anymore	2.57
		(1.72)

13	Because participation in my sport is consistent with my deepest principles	4.71
		(1.67)

14	For the satisfaction I experience while I am perfecting my abilities	5.00
		(1.41)

15	Because it is one of the best ways to maintain good relationships with my friends	5.29
		(1.49)

16	Because I would feel bad if I was not taking the time to do it	4.25
		(1.81)

17	It is not clear to me anymore; I do not really think my place is in sports	1.51
		(0.82)

18	For me, the pleasure of discovering new performance strategies	3.76
		(1.59)

19	For the material and/social benefits of being an athlete	4.54
		(2.01)

20	Because training hard will improve my performance	4.94
		(1.58)

21	Because participation in my sport is an integral part of my life	5.90
		(1.19)

22	I do not seem to be enjoying my sport as much as I previously did	2.32
		(1.44)

**Table 5:** Sport Motivation Scale (short form) mean score by six-factor subscale and Cronbach's alpha reliability score.

Sport motivation subscale	1st question	2nd question	3rd question	4th question	Mean	Cronbach's alpha
Amotivation (Q. 5, 12, 17, and 22)	1.94	2.57	1.51	2.32	2.09	0.75
External regulation (Q. 4, 11, and 19)	3.83	3.46	4.54		3.94	0.61
Introjected regulation (Q. 7, 10, and 16)	4.30	4.59	4.25		4.38	0.68
Identified regulation (Q. 3, 8, 15, and 20)	4.06	4.17	4.25	4.94	4.36	0.61
Integrated regulation (Q. 2, 9, 13, and 21)	5.56	5.25	4.71	5.90	5.36	0.80
Intrinsic motivation (Q. 1, 6, 14, and 18)	5.76	4.83	5.00	3.76	4.84	0.73
Total						0.84

**Table 6:** Nutritional themes around food intake during lockdown.

Themes	Subthemes	Example quotations
Improvement/deterioration (opportunity)	Time	*“In regard to timing, I am still eating at similar times.”*
		*“I'm in better physical and nutritional shape in lockdown due to extended time to cater for myself”*
		*“More time to cook. No hockey training or socialising means meals can be regular and planned”*
		*“Eat less, at more consistent times each day (3 meals a day at regular times) and balance of diet improved but eating more”*
		*“Consistency and always home-cooked”*
		*“Taking more care to eat healthier options and at regular intervals. Drinking alcohol in much lower quantities”*
	Quality	*“Eating more at home has led to cutting out on processed meals”*
		*“More fresh produce, more time to cook from scratch”*
		*“Improved in terms of nutrition and variety”*
		*“I'd say the quality has got better as has the experience of making it. More time in the kitchen with my wife creating new habits”*
		*“Lower quality lunches. More evening snacks”*

Food access (opportunity)	Emotional eating	*“Boredom eating, finding shopping for things irritating so picking up snacks etc. as a reward”*
		*“I have become much more aware of what I'm eating and often not in a very health (sic) way”*
		*“Comfort eating. More chocolate*
	Diet adaptation	*“I'm vegan and at points certain foodstuff were hard to get hold of I felt I should not go to multiple shops and so I curtailed my normal buying habits”*
		*“Changed to a vegan diet and eating at more regular times”*
		*“Major change is that I have started taking creatine preworkout before gym sessions, and drinking protein shakes after gym sessions and eating more protein bars”*

**Table 7:** Performance themes around physiological changes during lockdown.

Themes	Subthemes	Example quotations
Adaptation (motivation)	Fitness levels	*“Training sessions are a lower intensity and shorter. Less varied therefore found it difficult to stick to a regular routine”*
		*“Training on my own has been a huge struggle, because I really missed the motivation of my teammates”*
		*“Generally, I have got fitter. I enjoy running and training for a challenge, so I ran my first half marathon, my fastest 5 k (17.30 if you are interested) and equalled by 10 kpb”*

Physiological impact (capability)	Body composition	*“Decline in muscle mass and increase in fat. Higher alcohol consumption and less exercise”*
		*“The inability to go to the gym and do weight training has resulted in less muscle”*
		*“Lost 7 kgs from eating better but less muscle given no gyms are open”*
		*“Weight is 10% higher than normal”*
	Energy levels	*“Energy levels have dropped and exercising has been a battle”*
		*“WFH and not really leaving the house made me much more lethargic”*
		*“More often felt demotivated, less energetic, concentration span declined”*
	Cognitive focus	*“Feel more energised and focus. Have become slimmer but probably put on a bit more muscle”*
		*“In the beginning there was a constant feeling of exhaustion. Now with intermittent fasting I feel like I focus so much better and I can choose when to dedicate that time to eat. Instead of just doing it constantly or when I get a craving”*
	Sleep patterns	*“I have toned up quite a lot and feel more energetic—although I am also getting more sleep from not commuting so that will be helping”*
		*“I sleep late was furloughed so lost a lot of structure, struggling to get regular sleeping pattern back”*
		*“My sleep patterns have typically been consistent at around 7 hours. However,…the stress of working in ICU made it difficult to sleep more than 5 hours”*

**Table 8:** Mental health themes around behavioural change during lockdown.

Themes	Subthemes	Example quotations
Social restriction (opportunity)	Self-criticism	*“I started to not be so hard on myself, to understand the situation and try to keep trying. Though it is been difficult I am still trying to get my fitness up and my nutrition and health in order. Previously I would have just given up”*
		*“Hockey is really an outlet for me to spend time with friends to achieve a common goal. Not being able to do that led to me being much more self-critical than usual”*
		*“I am very hard on myself and need to be kinder to myself”*
		*“I need sports and face-to-face social interaction to properly function”*

Working from home (opportunity)	Behaviour change	*“Big increase in screen-time and much missed social interaction”*
		*“More mindful about not eating as much processed sugar. I have also started daily morning yoga which has improved my mental health massively”*
		*“More weight training, less junk food eaten, and less alcohol consumed”*
		*“Drank more water. Started practicing (sic) yoga. Learned more about myself and what feels good in my body and doesn't”*
		*“Too much time to think, loss of social interaction”*
		*“Far more sedentary and a reliance on food for short-term mood boosts”*
		*“Worked longer hours with working from home, meaning less movement and less work life balance”*

Coach support (motivation)	Communication	*“Nothing more…considering the difficult circumstances with the pitch redevelopment etc. they did a good job”*
		*“Relay information as and when received to update on the state of play”*
		*“Physically and tactically, we were very well positioned for Return to Play. We had a fantastic season”*
		*“Adolescent growth is one of the main reasons to abandon sport in this age for men, they grow every day, so their skills are affected—lockdown definitely made this worse”*

Club support (motivation)	Communication	*“Not much, the coach is an extension of the club, so I feel I have been well supported”*
		*“The hockey club was fantastic in keeping us informed about what was happening and ran online coaching sessions and information evenings”*

**Table 9:** Coach reflections.

Themes	Subthemes	Example quotations
Role change (motivation)	Focus	*The most important thing was to cheer all the players and make them integrated to the routine again”*
		*“We focussed on providing player feedback and nominating the players with the most potential to the next level of the Player Pathway (Performance Centre)”*
		*“I think the big and most important change was the motivation factor”*

Team contact (motivation)	Making the most of it	*“Once restrictions allowed, we ran a series of summer training sessions. This involved designing around 20 new socially distanced exercises—the aim was “to make the most of our time on the hockey pitch”. To reduce pressure, we made it clear that their performance, at this stage, had no impact on selection”*
		*“It is a bit difficult for an adult coach to establish direct contact with a junior player, so our approach was for the coaches to make contact with all the parents and for a WhatsApp group to be created. Over the various lockdowns we had skills challenges, fitness challenges, a Strava group and we ran a detailed player survey and discussed it over Zoom”*
		*“I think motivation was very high, they really missed hockey”*

Return to play (opportunity)	Mental health	*“Mentally I feel like it took a bit longer for some of the players to return to something like normal”*
		*“After lockdown the players were as combative as ever. Perhaps sport gave them the outlet they required”*
	Physical fitness	*“Physically and tactically, we were very well positioned for Return to Play. We had a fantastic season”*
		*“Adolescent growth is one of the main reasons to abandon sport in this age for men, they grow every day, so their skills are affected—lockdown definitely made this worse”*

## Data Availability

The datasets generated and/or analysed during the current study are not publicly available but are available from the corresponding author on reasonable request.
